# Enhanced UnaG With Minimal Labeling Artifact for Single-Molecule Localization Microscopy

**DOI:** 10.3389/fmolb.2021.647590

**Published:** 2021-04-20

**Authors:** Sangyoon Ko, Jiwoong Kwon, Sang-Hee Shim

**Affiliations:** ^1^Center for Molecular Spectroscopy and Dynamics, Institute of Basic Science, Seoul, South Korea; ^2^Department of Chemistry, Korea University, South Korea

**Keywords:** fluorescent protein, UnaG, photoswitching, super-resolution (SR) imaging, bilirubin, single-molecule localization microscopy

## Abstract

We introduced enhanced UnaG (eUnaG), a ligand-activatable fluorescent protein, for conventional and super-resolution imaging of subcellular structures in the mammalian cells. eUnaG is a V2L mutant of UnaG with twice brighter bulk fluorescence. We previously discovered the reversible fluorescence switching behavior of UnaG and demonstrated the high photon outputs and high localization numbers in single-molecule localization microscopy (SMLM). In this study, we showed that the fluorescence of eUnaG can be switched off under blue-light illumination, while a high concentration of fluorogenic ligands in the buffer can efficiently restore the fluorescence, as in UnaG. We demonstrated the capacity of eUnaG as an efficient protein label in mammalian cells, as well as for SMLM by utilizing its photoswitchable nature. While cytosolic UnaG proteins showed aggregated patches and fluorescence reduction at high expression levels, eUnaG-labeled protein targets successfully formed their proper structures in mammalian cells without notable distortion from the endogenous structure in the majority of transiently expressing cells. In particular, eUnaG preserved the vimentin filament structures much better than the UnaG. eUnaG provided similarly high single-molecule photon count distribution to UnaG, thus also similarly high resolution in the super-resolution images of various subcellular structures. The sampling coverage analysis of vimentin filaments in SMLM images showed the improvement of labeling efficiency of eUnaG. eUnaG is a high-performance fluorescent protein for fluorescence and single-molecule localization imaging in green emission with minimal labeling artifact.

## Introduction

Fluorescent proteins (FPs) are workhorses in live-cell fluorescence microscopy due to the facile and specific labeling of the target proteins (Chudakov et al., 2010; Rodriguez et al., 2017). Likewise, FPs have been extensively used in single-molecule localization microscopy (SMLM) (Zhou and Lin, 2013; Shcherbakova et al., 2014; Sauer and Heilemann, 2017). The first demonstrations of SMLM used photoactivatable fluorescence proteins (Betzig et al., 2006; Hess et al., 2006) along with organic dyes (Rust et al., 2006; Sharonov and Hochstrasser, 2006). The on–off transition of fluorescence emission is required in the SMLM for the temporal separation of individual molecules within the diffraction-limited area, allowing high precision localization of the individual molecules. The quality of the resultant super-resolution image is determined by two photophysical characteristics of the fluorophores. The photon number emitted from the fluorescent state determines the localization precision of determining the centroid position of a single fluorophore (Thompson et al., 2002). The number of switching cycles and the fraction of time spent in the fluorescent state, termed as the on–off duty cycle, are related to the labeling density and the Nyquist resolution (Shroff et al., 2008). Most of the FPs offer lower photon numbers than the organic fluorophores, hence resulting in lower spatial resolutions (Patterson and Lippincott-Schwartz, 2002; Subach et al., 2009; Brakemann et al., 2011). Furthermore, FPs often show irreversible fluorescence transition that restricts the spot density or transition between two different emission states that complicates multicolor applications (McKinney et al., 2009).

Recently, we introduced UnaG protein as an efficient SMLM probe for multicolor live-cell imaging (Kwon et al., 2020). UnaG is a ligand-activatable FP with a fluorogenic ligand, bilirubin (Kumagai et al., 2013). The ligand and apoUnaG protein are non-fluorescent in solution and become fluorescent upon binding to form holoUnaG. Photoswitching of UnaG is mediated by repetitive binding of bilirubin after photooxidation of the ligand followed by detachment of the damaged bilirubin from the protein. UnaG offers the highest photon numbers among blue-absorbing FPs and easily controllable switching kinetics. The off-switching rate is controlled by excitation intensity and oxygen concentration. The on-switching rate is linear to the concentration of bilirubin, whose fluorescence is undetectable up to micromolar concentration for supporting the repetitive recovery of fluorescence.

Here we investigated the photoswitching nature of enhanced UnaG (eUnaG) protein and its application to SMLM. eUnaG is a single-mutated variant of UnaG, in which Val 2 is substituted with leucine (Yeh et al., 2017). The single mutation near the N-terminus boosts the brightness of bacterial expression to about twofolds (Yeh et al., 2017). Since UnaG is a suitable fluorescent protein for SMLM, we measured the single-molecule photophysical characteristics of eUnaG and explored the SMLM imaging capability. We found that lower aggregation tendency of eUnaG leads to the benefit in the morphology and brightness of cellular structures, especially in the case of vimentin filament, whose morphology is sensitive to the fused tags.

## Materials and Methods

### Plasmids

Genetic incorporation using standard restriction enzymes and T4 DNA ligase was performed to construct eUnaG plasmids. eUnaG mutation (V2L) was performed *via* QuikChange site-directed mutagenesis protocol on the UnaG-mCherry construct. eUnaG-mCherry construct was utilized for PCR template for other eUnaG constructs. The primers for PCR amplification contain the sequences for small epitope tags such as HA or Flag. PCR products were digested with restriction enzymes and ligated into cut vectors (e.g., pcDNA3 and pDisplay). For the expression in mammalian cells, the CMV promoter was introduced in all cases. More detailed information about the genetic constructs is summarized in Supplementary Table 1.

### Cell Culture, Transfection, and Fixation

Cos-7 cells [Korean Collection for Type Cultures (KCTC)] were grown in Dulbecco’s modified Eagle’s medium (DMEM, SH30022.01, Hyclone, UT, United States) containing 10% v/v fetal bovine serum (97068-085, VWR Life Science, PA, United States) and 1% v/v antibiotic-antimycotic (15240-062, Gibco, MD, United States). The cells were electroporated (MPK5000, Invitrogen, CA, United States) during the general subculture, with ∼500 ng of proper plasmid, and seeded on a coverslip-bottomed 8-well chamber (155409, Lab-Tek, MI, United States) at the density of 5 × 10^4^ per well. After 24–48 h of transfection, the cells were fixed in 3% paraformaldehyde (50-980-495, Electron Microscopy Sciences, PA, United States) with 0.2% glutaraldehyde (16020, Electron Microscopy Sciences, PA, United States) for 10 min at room temperature.

### Imaging Buffers

Photon statistics and super-resolution imaging of eUnaG were performed in imaging buffer based on Tris pH 8 (10 mM, TRI01, LPS Solution, Daejeon, South Korea) containing NaCl (50 mM, 7548-4405, Daejung, Siheung, South Korea) and β-D-glucose (10% v/v, G7021, Sigma, MD, United States). To reduce the concentration of dissolved oxygen, we added an enzyme-based oxygen scavenging system (GLOX) containing glucose oxidase (560 μg/ml, G2133, Sigma, MD, United States) and catalase (400 μg/ml, C100, Sigma, MD, United States); 1 μM of external bilirubin (B4126, Sigma, MD, United States) was supplied to the imaging buffer during the SMLM imaging.

### Fluorescence Microscope

**FIGURE 1:**
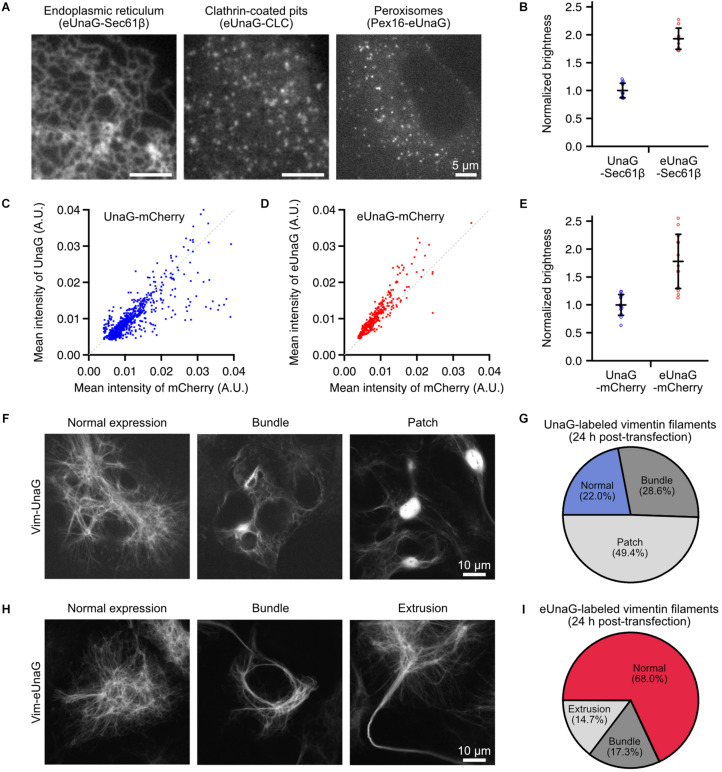
Mammalian expression of eUnaG-labeled protein in Cos-7 cells. **(A)** Widefield images of eUnaG-labeled Sec61β, CLC, and Pex16 proteins for visualization of the endoplasmic reticulum, clathrin-coated pits, and peroxisomes, respectively. **(B)** Fold increase in the fluorescence intensities of eUnaG [red dots, *n* = 10 fields of view (FOVs)] compared to those of UnaG (blue dots, *n* = 11 FOVs) measured from UnaG-Sec61β fusion proteins. Each data point is the mean intensity of the entire FOV of a confocal fluorescence image containing tens of cells. **(C,D)** Fluorescence intensity distribution at various expression levels of cytosolic UnaG-mCherry **(C)** and eUnaG-mCherry **(D)** fusion proteins. Each data point was measured from a cell detected from a large-FOV confocal image by CellProfiler (*n* = 803 for UnaG, *n* = 459 for eUnaG). The gray dotted line is drawn along *y* = *x* in **(C,D)**. **(E)** Fold increase in the fluorescence intensities of eUnaG (red dots, *n* = 13 FOVs) from those of UnaG (blue dots, *n* = 13 FOVs) measured from confocal images of Cos7 cells expressing cytosolic UnaG-mCherry proteins. The fluorescence intensities from UnaG and eUnaG proteins were normalized to the mCherry fluorescence intensities. Each data point is the mean intensity of the entire FOV containing tens of cells. **(F)** Confocal images of the expression pattern of UnaG-labeled vimentin filaments in Cos-7 cells. The cells were fixed 24 h after the transfection. **(G)** Phenotype distribution of cells expressing Vim-UnaG quantified from **(F)** (*n* = 91 cells). **(H)** Example confocal images of the expression pattern of eUnaG-labeled vimentin filaments. The cells were fixed 24 h after the transfection as in **(F)**. **(I)** Phenotype distribution of the expression pattern of Vim-eUnaG proteins obtained from **(H)** (*n* = 75 cells). Error bars: SDs.

A commercially available spinning-disk confocal system (DragonFly, Andor, Belfast, United Kingdom) was used to acquire the confocal images of vimentin filament to compare the expression pattern (Figures 1F,H). A confocal-based multi-acquisition system, TCS SP8 from Leica with a TCSPC (HydraHarp 400, PicoQuant, Berlin, Germany), was used to measure the fluorescence lifetimes. Photon statistics, widefield, and SMLM images were obtained using a home-built imaging system; 405-, 488-, 561-, and 647-nm laser light sources (405 nm: 200 mW, OBIS, Coherent, CA, United States; 488 nm: 150 mW, OBIS, Coherent, CA, United States; 561 nm: 150 mW, OBIS, Coherent, CA, United States; 647 nm: 120 mW, OBIS, Coherent, CA, United States) coupled with a single-mode optical fiber (3.4 μm diameter, PM-488-FC, Thorlabs, NJ, United States) were collimated, expanded about 25 times, and delivered into a microscope (Eclipse Ti-E, Nikon, Tokyo, Japan) through a lens (*f* = 400 mm, AC508-200-A-ML, Thorlabs, NJ, United States) and a dichroic mirror (ZT405/488/561/647rpc, Chroma, VT, United States) to make widefield illumination on the sample plane in epi geometry. The fluorescence was collected by an objective lens (Plan Apo TIRF, 100×, NA 1.49, oil, Nikon, Tokyo, Japan) and filtered by an emission filter (ZET405/488/561/647m-TRF, Chroma, VT, United States). A focus maintaining system embedded in the microscope precisely kept the focal plane during the measurement. A back-illuminated scientific CMOS camera (sCMOS, Prime95b, Photometrics, AZ, United States) collected the filtered fluorescence signal through the tube lens, resulting in an image pixel size of 110 nm. The acquisition parameters were set to 12-bit sensitive detection mode with no pre- and post-processing.

### Data Acquisition and Analysis Software

Fusion, a commercial acquisition software from Andor, was used to acquire confocal images from the DragonFly confocal microscope. SymPhoTime64 from PicoQuant was used to acquire and analyze the fluorescence lifetime. To measure the intensities of UnaG- and eUnaG-fused Sec61β and mCherry, several confocal images containing tens of cells were obtained and analyzed by CellProfiler, an open-source software for cell image processing. An open-source imaging software, μManager, was used to control the sCMOS camera and to acquire the single-molecule images (Edelstein et al., 2014). The raw single-molecule movies were processed by a high-density analysis ImageJ plugin, HAWK, to disentangle overactivated and overlapped fluorescence spots (Marsh et al., 2018). The processed single-molecule movies were analyzed by ThunderSTORM, an ImageJ plugin, to localize the centroid positions and reconstruct SMLM images (Ovesný et al., 2014). Sampling coverages of vimentin filaments were analyzed using the basic functions of ImageJ and a home-build MATLAB code, by referring to a previous report (Virant et al., 2018). We used the term “sampling coverage” instead of “labeling coverage” used in the study by Virant et al. (2018), which can give a false impression that it is calculated from the number of labeled copies and endogenous non-fluorescent copies of vimentin. Following the procedures mentioned in the study by Virant et al. (2018), from SMLM images, individual vimentin filaments were manually selected and straightened using ImageJ. The home-built MATLAB code binarized the result images and calculated the fraction of the covered area of the middle three pixels to calculate the sampling coverage.

## Results

### Enhanced UnaG Expression in a Mammalian Cell

Although UnaG protein has been used for imaging applications of mammalian cells such as local bilirubin quantification (Park et al., 2016), biomolecular interactions (To et al., 2016), and super-resolution visualization of subcellular structures (Kwon et al., 2020), there is no report that uses eUnaG in mammalian cells to the best of our knowledge. To demonstrate its potential to visualize the subcellular structures in mammalian cells, we fused eUnaG with various proteins such as Sec61β, Vim, clathrin light chain (CLC), Pex16, and cytosolic mCherry; transiently expressed the fusion proteins in Cos-7 cells; and observed the expression patterns (Figure 1A and Supplementary Figure 1). All the eUnaG-labeled proteins formed their respective subcellular structures, without notable distortion in their structures for the majority of cells. When we directly compare the fluorescence intensity from UnaG- and eUnaG-labeled Sec61β proteins, eUnaG showed ∼2 times brighter signals (Figure 1B).

To normalize the fluorescence intensities by the expression levels varying in transiently expressing cells, we expressed the cytosolic mCherry fused with UnaG or eUnaG. At the level of individual cells, the UnaG intensity was largely proportional to the mCherry intensity (Figure 1C). However, a noticeable portion of cells expressing UnaG-mCherry showed deviation from the linear dependence when the mCherry intensity was high (Figure 1C). In contrast, the eUnaG intensity was mostly linear to the mCherry intensities with few exceptions (Figure 1D). Also, the normalized intensities of eUnaG to mCherry appear to be slightly higher than those of the linear fraction of UnaG. Assuming that the mCherry intensity is proportional to the concentration of the fusion protein molecules in each transiently expressing cell, these results can be interpreted as the concentration-dependence of the fluorescence intensities of UnaG and a slight increase of eUnaG fluorescence. The high concentration of cytosolic UnaG could induce UnaG-mediated protein aggregation, resulting in loss of fluorescence *via* aggregation-induced quenching or inhibition of ligand binding. Since the fluorescence lifetimes of UnaG and eUnaG are very similar under the same measurement condition (Supplementary Figure 2), this aggregation-induced reduction of UnaG fluorescence could contribute to the bulk brightness difference between UnaG and eUnaG. The ensemble fluorescence intensities of UnaG and eUnaG from cytosolic UnaG- and eUnaG-mCherry fusion proteins showed approximately twofold enhancement of eUnaG fluorescence, when normalized by mCherry signal (Figure 1E). This is consistent with the result from Sec61β fusion proteins (Figure 1B) and the previous result from bacterial expression (Yeh et al., 2017).

To compare the degree of aggregation, we examined the induced formation of stacked ER membrane whorls, termed OSER (organized smooth ER), which are commonly used for the characterization of the oligomerization levels of FPs (Costantini et al., 2012). We compared UnaG-Sec61b and eUnaG-Sec61b OSERs to mMaple3-Sec61b OSERs (Supplementary Figure 3). Previously, mMaple3 was classified as monomeric by counting OSER in CytERM-mMaple3 fusions (Kaberniuk et al., 2018). In our Sec61b-based assays, UnaG and eUnaG showed a lower level of oligomerization than mMaple3 (Supplementary Figure 3), indicating the monomeric character of UnaG and eUnaG. Also, eUnaG showed a slightly lower level of aggregation than that of UnaG, supporting the possibility of aggregation-induced reduction of fluorescence.

We further examined the expression pattern of vimentin filaments labeled with UnaG and eUnaG. Vimentin filaments have been used as a reference structure for evaluating labeling artifacts because the cellular structure is sensitive to the multimeric character of the fused FPs (Wang et al., 2014). Most of the FPs for SMLM are mutants of multimeric FPs, and the residual tendency to multimerize can induce abnormally thick fiber bundles of vimentin filament (Wang et al., 2014). For instance, mMaple3, considered as monomeric by OSER (Kaberniuk et al., 2018), showed ∼30% of cells with normal vimentin filaments and ∼70% of cells with abnormal phenotypes (Supplementary Figure 4). When we transiently expressed UnaG-labeled vimentin for 24 h, only ∼20% of fluorescent cells showed normal vimentin structures of thin filaments (Figures 1F,G and Supplementary Figure 5), and the remaining cells showed abnormal expression patterns such as bundled filaments (“Bundle” in Figure 1F) and bright fluorescent patches (“Patch” in Figure 1F). The “Patch” structures are consistent with the non-linear behavior of UnaG intensities at high expression level (Figure 1C). These abnormal structures may stem from the aggregation of Vim-UnaG proteins in high cytosolic concentrations. When observing the phenotypes after 4 days of electroporation, ∼65% of UnaG-expressing cells showed normal vimentin distribution (Supplementary Figure 6), indicating that low expression levels help to prevent aggregation from cytosolic UnaG, while the Vim-UnaG proteins incorporated into the filaments did not perturb the filamentous structure. In contrast, ∼70% of Vim-eUnaG expressing cells showed a normal expression pattern of thin vimentin filaments after 24 h from the transfection (Figures 1H,I and Supplementary Figure 5); ∼30% of Vim-eUnaG-expressing cells showed long, thick bundle of vimentin fibers as the major artifact. These bundled filaments were formed in either cytosolic area around the nucleus (“Bundle” in Figure 1H) or cell periphery forming extrusions reaching out of the cell (“Extrusion” in Figure 1H). Interestingly, Vim-eUnaG-expressing cells did not show “Patch” phenotype found in ∼50% of Vim-UnaG-expressing cells. This is consistent with the OSER results, indicating the lower aggregation tendency of eUnaG (Supplementary Figure 3). Thus, eUnaG can serve as a facile fluorescent marker for artifact-free imaging applications to visualize various subcellular structures in the mammalian cells by protein fusion and transient expression, without any severe distortions in the target structures for the majority of cells.

### Photoswitching Nature of eUnaG

**FIGURE 2:**
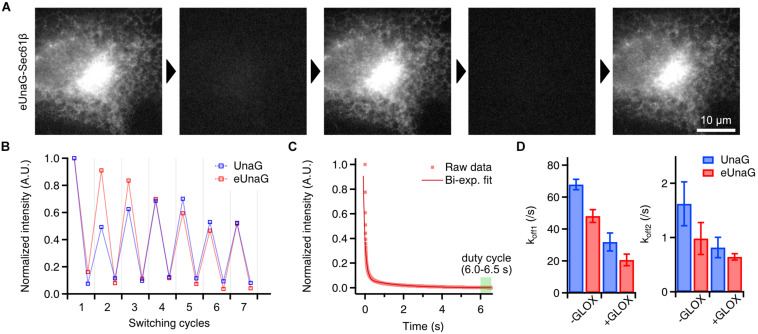
Photoswitching behavior of eUnaG. **(A)** Widefield images of a fixed eUnaG-Sec61β expressing Cos-7 cell showing repetitive fluorescence switching of eUnaG. Images were obtained from two successful rounds of fluorescence switching (off-switching: 300 W/cm^2^ of 488-nm laser for 20 s; on-switching: 1 μM bilirubin for 5 min). **(B)** Repetitive on- and off-switching of UnaG and eUnaG proteins. Fluorescence intensities were measured from a series of widefield images as in **(A)** and normalized to the initial value. **(C)** Fluorescence intensity trace of eUnaG under 300 W/cm^2^ of 488-nm laser illumination. The decaying profile was well fitted by a bi-exponential function, giving two off-switching rates, k_off1_ and k_of__f2_. The duty cycle was calculated from the average intensity at the equilibrium state (6.0–6.5 s). **(D)** Two off-switching rates of UnaG and eUnaG under different concentrations of dissolved oxygen (number of measurements: 5 for UnaG -GLOX, 14 for UnaG +GLOX, 6 for eUnaG -GLOX, 12 for eUnaG +GLOX). UnaG and eUnaG showed a similar tendency of slower off-switching rates with lower concentration of dissolved oxygen (+GLOX), in both k_off1_ and k_of__f2_. Error bars: SDs.

Since eUnaG is a single-residue mutant of UnaG at the N-terminus, we postulated that the fluorescence of eUnaG can be switched off by light illumination and turn back on by rebinding with the fluorogenic ligand, bilirubin, like UnaG. We tested the photoswitching behavior of eUnaG with eUnaG-Sec61β expressing Cos-7 cells (Figure 2A). Upon a high-intensity illumination of 488-nm laser light (300 W/cm^2^), the fluorescence from eUnaG quickly bleached off within 10 s. When we treated the bleached eUnaG proteins with 1 μM of exogeneous bilirubin, the fluorescence recovered in high efficiency, allowing the structure of the endoplasmic reticulum to be clearly visualized again. The on–off switching cycle could be repeated multiple times as observed in UnaG, and the recovery efficiency was quite similar to UnaG during the repetitive switching cycles (Figures 2A,B).

This common way of reversible switching indicates that the photoswitching mechanism of eUnaG is similar to that of UnaG. Indeed, when we measured the off-switching rate in different concentrations of dissolved oxygen, there were two different off-switching speeds (k_off1_ and k_off2_) as in UnaG, corresponding to the two fluorescent forms of UnaG: less bright conformation that forms immediately after binding to bilirubin, known as UnaG1; brighter conformation (UnaG2) that spontaneously converts from UnaG1 (Figure 2C; Shitashima et al., 2017; Kwon et al., 2020). Both the off-switching rates of k_off1_ and k_off2_ for eUnaG were highly dependent on the concentration of dissolved oxygen, implying the similarity of off-switching mechanism that is mediated by the photo-oxidation of bilirubin molecule under intense 488-nm illumination (Figure 2D).

From the fluorescence intensity trace under off-switching condition, we can measure the on–off duty cycle from the ratio of the equilibrium intensity to the initial, unbleached intensity (Dempsey et al., 2011). With 300 W/cm^2^ of illumination intensity, the duty cycle of eUnaG was measured as 0.0015 within ∼7 s, which is comparable to the best-performing SMLM dyes such as Alexa 647 (Figure 2C).

**FIGURE 3:**
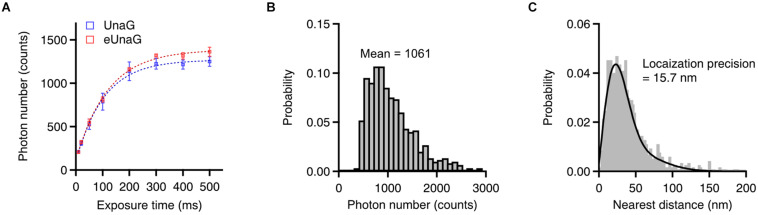
Single-molecule switching characteristics of eUnaG. **(A)** Measured photon counts with different exposure times (*n* = 8 independent measurements). The saturation levels of photon counts were 1,270 and 1,390 for UnaG and eUnaG, respectively, indicating a slightly higher photon number of eUnaG. **(B)** Photon number distribution of eUnaG with 200 ms of exposure time, showing 1,061 mean photon counts corresponding ∼22 nm of theoretical spatial resolution. **(C)** Resolution measurement by the nearest neighbor analysis yielded ∼16 nm of spatial resolution from repetitive localization of eUnaG. Error bars: SDs.

The number of photons during the on-time of each switching cycle determines the localization accuracy in SMLM (Thompson et al., 2002). The photon number per switching cycle of eUnaG was saturated at a slightly higher level than that of UnaG as the exposure time increased (Figure 3A, saturation level: 1,270 photons for UnaG; 1,390 photons for eUnaG). The slight increase in the photon numbers of the eUnaG is consistent with the slight increase in the slope of the eUnaG/mCherry intensity plot (Figures 1C,D) and the slight decrease in the off-switching rate (Figure 2D). The average photon number value was 1,061 counts with 200 ms of exposure time, corresponding to the theoretical localization accuracy of ∼22 nm (Figure 3B). We measured the localization accuracy by the nearest neighbor analysis and obtained 15.7 nm for eUnaG, which is similar to the value obtained for UnaG (Figure 3C; Kwon et al., 2020). Note that the photon number of UnaG was on par with mMaple3, one of the brightest fluorescent proteins for SMLM (Wang et al., 2014; Kwon et al., 2020). With the even higher photon count of eUnaG, we can expect further resolution enhancement in SMLM images.

### Single-Molecule Localization Microscopy of eUnaG-Labeled Subcellular Structures

**FIGURE 4:**
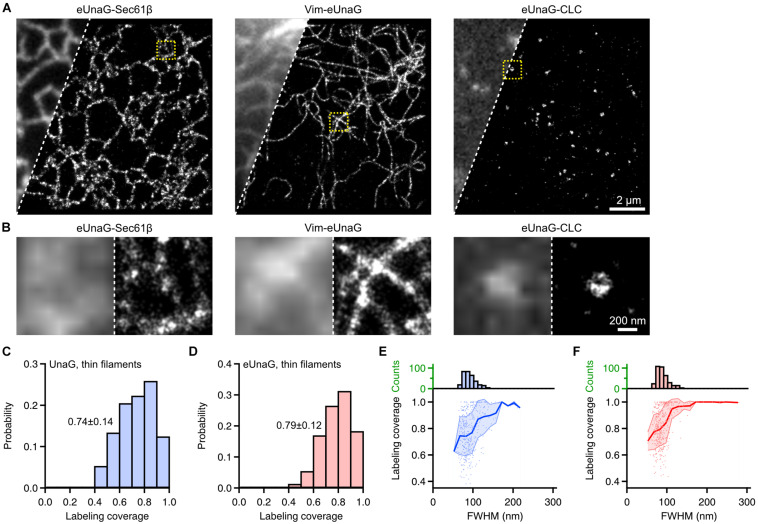
Single-molecule localization microscopy of eUnaG-labeled subcellular structures in fixed Cos-7 cells. **(A)** Widefield (left) and SMLM (right) images of Cos-7 cells transfected with eUnaG-Sec61β, Vim-eUnaG, and eUnaG-CLC. **(B)** Zoom-in images from yellow-dotted boxes in **(A)**. Each panel contains widefield (left) and SMLM (right) images. **(C,D)** Sampling coverage of thin vimentin filaments fused with **(C)** UnaG and **(D)** eUnaG, obtained from corresponding SMLM images. *n* = 102 and 180 filaments for UnaG and eUnaG, respectively. Displayed values: Mean ± SD. **(E,F)** Correlation between FWHM of vimentin filament and the sampling coverage for **(E)** UnaG- and **(F)** eUnaG-labeled vimentin filaments, with corresponding distribution of FWHM (upper histograms); 153 and 222 filaments were analyzed for UnaG and eUnaG, respectively. Shaded error regions indicate SDs at each FWHM.

As in UnaG, we obtained SMLM images of Sec61β, Vim, and CLC labeled with eUnaG (Figure 4). These images were obtained with 1 μM exogeneous bilirubin under widefield Epi illumination. In this condition, the fluorescence background was nearly unchanged since bilirubin is non-fluorescent in solution and becomes fluorescent only when bound to the protein (i.e., bilirubin is fluorogenic) (Kwon et al., 2020). Also, the on-switching rates and the accumulated localization number per SMLM image could be controlled by the bilirubin concentration. The combination of high photon number and high localization number from reversible, controllable switching of eUnaG allowed us to obtain high SMLM resolution that is determined by both localization precision (determined by photon number) and Nyquist resolution (determined by localization density) (Thompson et al., 2002; Shroff et al., 2008). The raw single-molecule movies were obtained for > 16,000 frames with 20 ms of exposure time and were analyzed by HAWK software to reduce the unwanted artifacts from overlapping single-molecule images (Supplementary Figure 7; Marsh et al., 2018). The side-by-side comparison of widefield and SMLM images showed the resolution enhancement by SMLM imaging, and the beneficial photophysical characteristics of eUnaG enabled clear visualization of the ring-like structure of clathrin-coated pits (Figure 4B). Also, the average width of vimentin filaments measured by the full width in half maximum (FWHM) was ∼57 nm (Supplementary Figure 8). The widths of vimentin filaments imaged with UnaG and eUnaG were broader than those with Alexa 647, a standard best-performing organic dye for SMLM, but narrower than those with mMaple3, one of the SMLM FPs with the highest photon numbers (Supplementary Figure 9). Since Alexa 647 was linked to vimentin through streptavidin and AviTag (a peptide tag composed of 12 amino acids), the linkage error (i.e., the distance between the target protein and the fluorophore) can be approximated as roughly the same with FP-fused fibrils. Regarding the ∼10-nm width of vimentin fibrils, the measured widths from SMLM images were consistent with the localization accuracies measured from the nearest neighbor analysis (NeNA) of vimentin SMLM images (Supplementary Figure 9), except for mMaple3 with low fiber sampling coverage (Endesfelder et al., 2014; Kwon et al., 2020).

Even though the fractions of cells with normal expression patterns differ dramatically for UnaG and eUnaG (Figures 1F–I), we could still find cells with normal vimentin structures and obtain SMLM images. From the SMLM images of vimentin fibers, we quantified the fiber sampling coverage calculated from the longitudinal fraction of fibers sampled by the localization points (Figures 4C–F and Supplementary Figure 10; Virant et al., 2018). The sampling coverages for UnaG and eUnaG from cells with normal expression patterns were largely similar (Figures 4C,D). We also obtained a similar distribution of fiber widths and similar correlations of fiber FWHM to the sampling coverage of individual fibers for UnaG and eUnaG (Figures 4E,F). The fiber widths and sampling coverages of UnaG and eUnaG were on par with the pair of BC2 tag and nanobodies (Virant et al., 2018), indicating that both UnaG and eUnaG are incorporated into the vimentin fibrils at proper cytosolic concentrations. For both UnaG- and eUnaG-labeled fibrils, the sampling coverage increases as the width increases, approaching the complete sampling of fibrils exceeding a width of 200 nm (Figures 4E,F). However, the coverage for the thinnest fibers was slightly (∼10%) higher for eUnaG-labeled fibrils. Also, for widths in 100–200 nm, the coverage of eUnaG-labeled fibrils was plateaued to 100%, while the coverage of UnaG-labeled fibrils remained well below 100%. These differences contributed to a slightly higher mean value and a narrower deviation in the distribution of sampling coverage for eUnaG-labeled fibrils (Figures 4C,D). In addition, the bright UnaG patches found in many UnaG-expressing cells indicate the tendency of UnaG to aggregate at high cytosolic concentration (Figures 1F,G). Altogether, eUnaG provides a way for high-quality SMLM imaging in green emission channel with better expression patterns and lower labeling artifacts.

## Discussion

We demonstrated the capacity of eUnaG as a potential fluorescent marker for SMLM in the green emission channel. Like UnaG, eUnaG can undergo repetitive fluorescence switching cycles required for SMLM, and the on- and off-switching are mediated by the rebinding and photo-oxidation of bilirubin, respectively. A single eUnaG molecule offers a slightly higher photon number than UnaG, and thus, it can potentially provide the highest spatial resolution among the existing FPs for SMLM. In addition to the unique benefits in SMLM, the clear improvements in labeling artifact of eUnaG make it a much better probe for SMLM as well as conventional diffraction-limited imaging.

The slight increase in photon number (Figure 2) may appear inconsistent with the significant increase in bulk fluorescence intensity (Figures 1B,D). However, the population of UnaG with non-linear fluorescence intensity to the expression level (Figure 1C), the higher level of aggregation in OSER (Supplementary Figure 3), and the bright circular patches found in 49% of Vim-UnaG cells (Figures 1F,G and Supplementary Figure 5) support the hypothesis of aggregation-induced reduction of fluorescence. The similar lifetimes of UnaG and eUnaG (Supplementary Figure 2) rule out the possibility of aggregation-induced quenching, leaving aggregation-induced inhibition of ligand binding as a plausible cause. Restriction of access to ligands reduces the number of fluorescent UnaG molecules, but does not influence the single-molecule brightness.

Enhanced UnaG can easily label protein targets *via* standard genetic incorporation and transient expression in mammalian cells. eUnaG provides better expression patterns than UnaG, especially for proteins like vimentin whose structure can be easily perturbed by protein aggregation. Short peptide tags such as BC2 tag and ALFA tag have shown a similarly low level of labeling artifacts in vimentin fibers (Virant et al., 2018; Götzke et al., 2019). However, the fluorescent labeling of the tags requires delivery of fluorophore-conjugated nanobodies. For instance, anti-BC2 nanobody was delivered into live cells by protein transfection. In contrast, bilirubin, the small-molecule ligand, is cell-permeable and can be delivered into live and fixed cells by simply adding it to the culture medium (Kwon et al., 2020). Moreover, while the non-specifically bound fluorescent nanobodies can increase background fluorescence, the non-fluorescent bilirubin ligands only become fluorescent after binding to UnaG and exhibit negligible non-specific background fluorescence in conventional (Supplementary Figure 11) and SMLM imaging even under Epi-illumination (Kwon et al., 2020).

The lower labeling artifact of eUnaG may be attributed to the stability of the protein and the ligand. The thermal stability of eUnaG was higher than UnaG (Yeh et al., 2017). Also, the bilirubin ligand in eUnaG was more rigid than that of UnaG due to the stronger interaction network among the ligand, the protein, and more stably bound water molecules in the binding cavity that supplement more hydrogen bonds (Lee et al., 2018). The higher stability of eUnaG may help to decrease the aggregation tendency. This is reminiscent of our observation that 49% of Vim-UnaG-expressing cells contained a large patch of aggregated proteins (Figure 1F, “Patch”). These circular patches in the juxtanuclear regions have the characteristics of collapsed vimentin structures associated with aggresome (Supplementary Figure 12; Johnston et al., 1998). Aggresome formation indicates that overexpression of Vim-UnaG produced a large amount of misfolded proteins, making the cells to employ proteostasis processes (Kawaguchi et al., 2003). In contrast, such aggregated structure was not found in any Vim-eUnaG-expressing cell.

In fact, eUnaG fused to vimentin may become a valuable tool for investigating proteostasis. Since the discovery of vimentin’s presence at the aggresome (Johnston et al., 1998), many studies have used vimentin as a marker for aggresome formation. Recent studies have begun to unveil the function of vimentin at the aggresome (Morrow and Moore, 2020). For example, vimentin recruits proteasome machineries to the aggresome, thereby playing a key role in stem cell proteostasis (Morrow et al., 2020). eUnaG may help to resolve ultrastructures and dynamics of vimentin at the aggresome *in vivo*, thereby revealing the organismal implications in aging and cancer.

We anticipate that eUnaG’s suppressed labeling artifact and excellent SMLM capabilities would make it a new workhorse in live-cell SMLM imaging.

## Data Availability Statement

The raw data supporting the conclusions of this article will be made available by the authors, without undue reservation.

## Author Contributions

S-HS proposed and designed the idea of this work. SK prepared the biological samples, acquired and analyzed the confocal images, evidence of photoswitching, and kinetics information. JK acquired the widefield and SMLM images and analyzed the single-molecule characteristics and sampling coverage. All authors contributed to the writing and manuscript revision.

## Conflict of Interest

The authors declare that the research was conducted in the absence of any commercial or financial relationships that could be construed as a potential conflict of interest.
