# Supplementary material for: Enhanced UnaG With Minimal Labeling Artifact for Single-Molecule Localization Microscopy
Source: Front Mol Biosci. 2021 Apr 20;8:647590. doi: 10.3389/fmolb.2021.647590 (PMC8093618; doi:10.3389/fmolb.2021.647590)
Supplement: Supplementary file 1 [file Data_Sheet_1.docx]

Supplementary Material

# Supplementary Tables

# Supplementary Table 1. Summary of genetic constructs used in this work.

| **Name** | **Features** | **Promoter**  **/Vector** | **Forward Primer/Reverse Primer** | **Notes** |
| --- | --- | --- | --- | --- |
| eUnaG-mCherry | *NotI*-eUnaG-Flag-*EcoRI*-mCherry-*BamHI*-His6-Stop-*XhoI* | T7/ pET21a | cgacgataagaagctagcaATGCTGGAAAAATTTGTTGGTACGTGGA/  TCCACGTACCAACAAATTTTTCCAGCATtgctagcttcttatcgtcg | - V2L mutation was introduced via site-directed mutagenesis on the UnaG-mCherry construct (PMID: 31937765)  - Flag: DYKDDDDK |
| eUnaG-Sec61β | NotI-eUnaG-Flag*-*EcoRI*-*Sec61β-Stop-XhoI | CMV/ pcDNA3 | tagtaggcggccgccaccATGctgGAAAAATTTGTTGGTACGTGG/  tagtaggaattccttatcgtcgtcatccttgtagtcTTCGGTTGCACGCCGGT | - V2L mutation was introduced via site-directed mutagenesis on the UnaG-Sec61β construct (PMID: 31937765) |
| mMaple3-Sec61β | NotI-mMaple3-Flag*-*EcoRI*-*Sec61β-Stop-XhoI | CMV/ pcDNA3 | tagtaggcggccgccaccATGGTGAGCAAAGGCGAGGA/ gaattccttatcgtcgtcatccttgtagtcCTTATAGAGTTCGTCCATGCTGTCGG | - pVim-mMaple3 was a gift from Xiaowei Zhuang in Harvard Univ. (PMID: 24912163). |
| Vim-eUnaG | HindIII-Vim-Linker-Flag-NheI-eUnaG-Stop-XhoI | CMV/ pcDNA5* | cgacgataagaagctagcaATGCTGGAAAAATTTGTTGGTACGTGGA/  TCCACGTACCAACAAATTTTTCCAGCATtgctagcttcttatcgtcg | - V2L mutation was introduced via site-directed mutagenesis on the Vim-UnaG construct (PMID: 31937765)  - Linker: SGGSGGS |
| eUnaG-CLC | NotI-eUnaG-Flag-EcoRI-CLC-Stop-XhoI | CMV/ pcDNA3 | tagtaggcggccgccaccATGctgGAAAAATTTGTTGGTACGTGG/  tagtaggaattccttatcgtcgtcatccttgtagtcTTCGGTTGCACGCCGGT | - YFP-Clathrin was a gift from Xiaowei Zhuang (Addgene plasmid # 20921) |
| Pex16-eUnaG | HindIII-Pex16*-* KpnI-eUnaG-Flag-Stop-*NotI* |  | TAGTAGGGTACCATGctgGAAAAATTTGTTGGTACG/ CTCGAGCGGCCGCCTActtatcgtcgtcatccttgtagtcTTCGGTTGCACGCCGGTAC | - PEX16 (NM_004813.2) |

* Due to the limitation in restriction sites, vimentin plasmids were prepared from pcDNA5 instead of pcDNA3.

# Supplementary Table 2. Widefield imaging parameters.

| **Figure** | **Microscope** | **Objective lens** | **Field of view (µm^2^)** | **Exposure time (ms) / frames** | **Excitation laser (nm)** | **Imaging buffer** |
| --- | --- | --- | --- | --- | --- | --- |
| Fig. 1A | Home-built STORM setup on Ti-E | Plan Apo  100x  TIRF  NA 1.49 | 16.5×16.5 for Sec61β and CLC  33.0×33.0 for Pex16 | 200 / 1 | 488 | UnaG IB* |
| Fig. 2A,B | Home-built STORM setup on Ti-E | Plan Apo  100x  TIRF  NA 1.49 | 33.0×33.0 | 1,000 / 1 | 488 | UnaG IB |
| Fig. 2C,D | Home-built STORM setup on Ti-E | Plan Apo  100x  TIRF  NA 1.49 | 16.5×16.5 | 5 / 2,000 | 488 | UnaG IB |
| Fig. 4A,B | Home-built STORM setup on Ti-E | Plan Apo  100x  TIRF  NA 1.49 | 11.0×11.0 for large FOV  1.0×1.0 for zoom-ins | 200 / 1 | 488 | UnaG IB |
| Fig. S1 | Home-built STORM setup on Ti-E | Plan Apo  100x  TIRF  NA 1.49 | 16.5×16.5 | 200 / 1 | 488 | UnaG IB |
| Fig. S11 | Home-built STORM setup on Ti-E | Plan Apo  100x  TIRF  NA 1.49 | 132×132 | 200 / 1 | 488 | UnaG IB (no bilirubin for “without bilirubin” condition) |

# * UnaG imaging buffer (IB): 10 mM Tris pH8.0, 50 mM NaCl, 10% v/v glucose, 560 µg/mL glucose oxidase, 400 µg/mL catalase, 1 µM bilirubin.

# Supplementary Table 3. Confocal imaging parameters.

| **Figure** | **Microscope** | **Objective lens** | **Field of view (µm^2^)** | **Acquisition time per image (ms)** | **Excitation laser (nm)** | **Imaging buffer** |
| --- | --- | --- | --- | --- | --- | --- |
| Fig. 1B-E | DragonFly  Confocal  Single slice | Plan Fluor  10x  NA 0.30 | 1,331×1,331 | 500 | 488 for UnaG/eUnaG  561 for mCherry | UnaG IB* for UnaG/eUnaG |
| Fig. 1F-I  Fig. S3-S6  Fig. S12 | DragonFly  Confocal  Single slice | Plan Apo  60x  NA 1.20 | 205.0×205.0 | 500 | 405 for DAPI  488 for UnaG/eUnaG and mMaple3  647 for Alexa 647 | UnaG IB for UnaG/eUnaG  PBS for mMaple3 |
| Fig. S2 | TCS SP8 | Plan Apo  63x  NA 1.40 | 184.5×184.5 | 1,364 per confocal image  ~30,000 per FLIM image | 488 | UnaG IB |

# * UnaG imaging buffer (IB): 10 mM Tris pH8.0, 50 mM NaCl, 10% v/v glucose, 560 µg/mL glucose oxidase, 400 µg/mL catalase, 1 µM bilirubin.

# Supplementary Table 4. SMLM imaging parameters.

| **Figure** | **Microscope** | **Objective lens** | **Field of view (µm^2^)** | **Exposure time (ms) / frames** | **Excitation laser (nm)** | **Imaging buffer** |
| --- | --- | --- | --- | --- | --- | --- |
| Fig. 3A,B | Home-built STORM setup on Ti-E | Plan Apo  100x  TIRF  NA 1.49 | 16.5×16.5 | Varied in A, 200 for B / 1,000 | 488 | UnaG IB* |
| Fig. 3C | Home-built STORM setup on Ti-E | Plan Apo  100x  TIRF  NA 1.49 | 4.0×2.6 | 20 / 16,384 | 488 | UnaG IB |
| Fig. 4  Fig. S7-S10 | Home-built STORM setup on Ti-E | Plan Apo  100x  TIRF  NA 1.49 | 11.0×11.0 for large FOV (Fig. 4A)  1.0×1.0 for zoom-ins (Fig. 4B)  16.5×16.5 for Fig. S7 and S9 | 20 / 16,384 | 488 for UnaG/eUnaG  405 and 561 for mMaple3 activation and excitation, respectively  647 for Alexa 647 | UnaG IB for UnaG/eUnaG  PBS for mMaple3  STORM IB** for Alexa 647 |

# * UnaG imaging buffer (IB): 10 mM Tris pH8.0, 50 mM NaCl, 10% v/v glucose, 560 µg/mL glucose oxidase, 400 µg/mL catalase, 1 µM bilirubin. ** STORM imaging buffer (IB): 10 mM Tris pH8.0, 50 mM NaCl, 10% v/v glucose, 560 µg/mL glucose oxidase, 400 µg/mL catalase, 100 mM MEA (β-mercaptoethylamine).

# Supplementary Figures

**Supplementary Figure 1.** Widefield images of UnaG- and eUnaG-labeled Sec61β, Vim, CLC and Pex16 proteins for visualization of the endoplasmic reticulum, vimentin filament, clathrin-coated pits, and peroxisomes, respectively.

**Supplementary Figure 2.** Fluorescence lifetime measurement of UnaG and eUnaG proteins transiently expressed in Cos-7 cells. Cytosolic UnaG-mCherry and eUnaG-mCherry fusion proteins were used for lifetime measurement. **(A)** Example TCSPC intensity trace of eUnaG protein, which was well fitted with a bi-exponential function. **(B)** Mean lifetime values of UnaG and eUnaG from 5 independent measurements (Error bars: standard deviations). UnaG and eUnaG showed highly similar lifetimes for both short and long components.


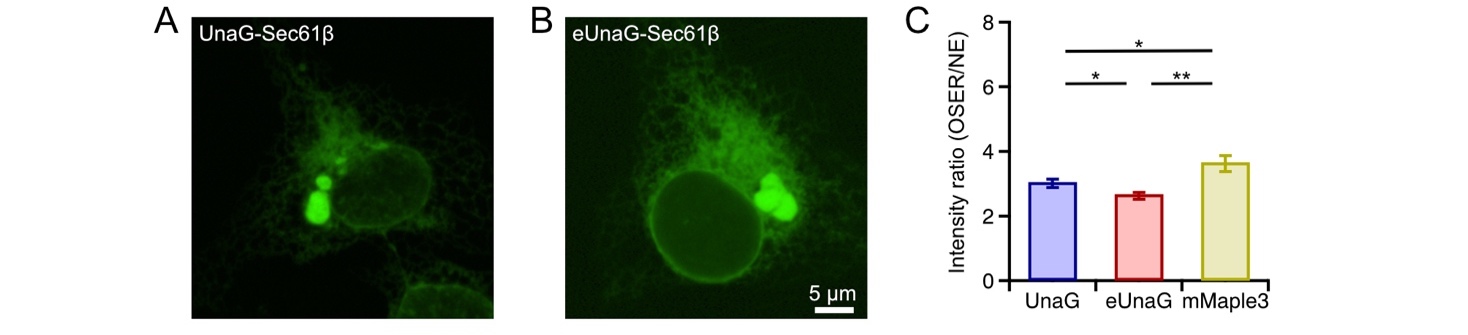


**Supplementary Figure 3.** Organized smooth endoplasmic reticulum (OSER) assay for comparison of aggregation level of UnaG, eUnaG and mMaple3 fused with Sec61β. FPs are fused at the N-termini of Sec61β to express the FPs at the cytosolic side of the ER. Cos-7 cells were transfected under the same electroporation conditions. (**A**,**B**) Example fluorescence images of OSER whorls observed in UnaG- and eUnaG-Sec61β expressing cells. In 349 cells expressing UnaG fusions (**A**), 91 OSER whorls were found in 40 cells. In 362 cells expressing eUnaG fusions (**B**), 99 OSERs were observed in 49 cells. (**C**) Intensity ratios of the whorl (OSER) structures over the mean intensity of the nuclear envelope (NE). The error bars represent standard errors. The p-values are 0.0343, 0.0290, and 0.0007 for UnaG-eUnaG, UnaG-mMaple3, and eUnaG-mMaple3, respectively.


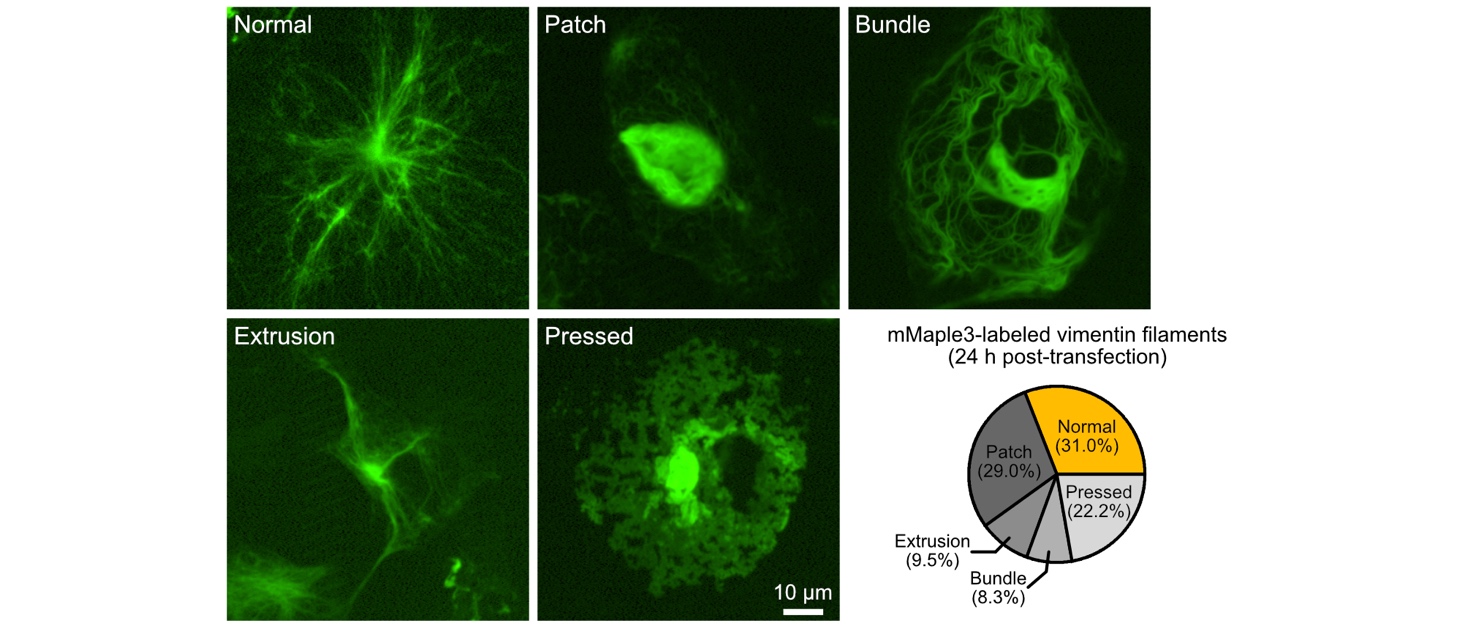


**Supplementary Figure 4.** Expression patterns of Vim-mMaple3 expressing Cos-7 cells after one day from transfection. (**A**) Example confocal images of mMaple3-labeled vimentin filaments for various phenotypes indicated in the top left corner of each image. (**B**) Phenotype distribution of Vim-mMaple3 proteins obtained from **A** (n = 252 cells).

**Supplementary Figure 5.** Examples of vimentin expression patterns of Cos7 cells expressing (**A**) UnaG-Vim and (**B**) eUnaG-Vim in addition to Figures 1F and 1H, respectively. The phenotype assignments are the same with Figures 1F and 1H.

**Supplementary Figure 6.** Expression pattern of UnaG-Vim expressing Cos-7 cells after one day (left) and four days (right) from transfection. Abnormal cells include both “Bundle” and “Patch” phenotypes in Figures 1F and 1G. After 4 days, more than 65% of cells showed normal expression pattern for the vimentin filament (n = 91 and 61 cells for 1 and 4 days, respectively).


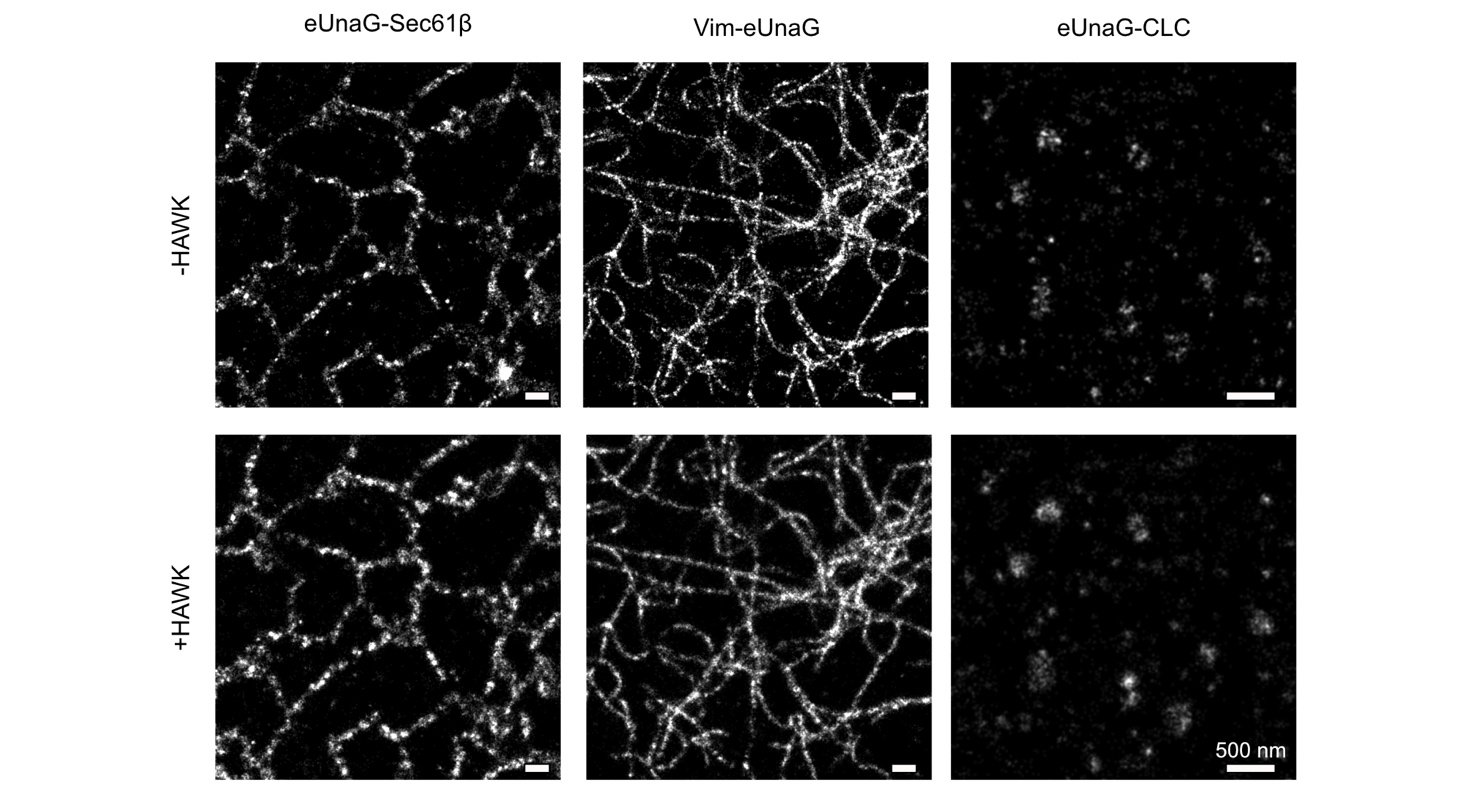


**Supplementary Figure 7.** Influence of HAWK analysis to the SMLM images of eUnaG fusions. Top: SMLM images analyzed with standard ThunderSTORM analysis without HAWK pre-processing. Bottom: SMLM images after processing with HAWK, followed by ThunderSTORM analysis.

**Supplementary Figure 8.** Intensity line profiles of vimentin filaments in SMLM images obtained in +HAWK condition. The intensity profiles (dotted lines) and their corresponding Gaussian-fits (solid lines) were displayed from 10 vimentin filaments. The average FWHM from the above 10 examples is 57±7 (mean±s.d.) nm.


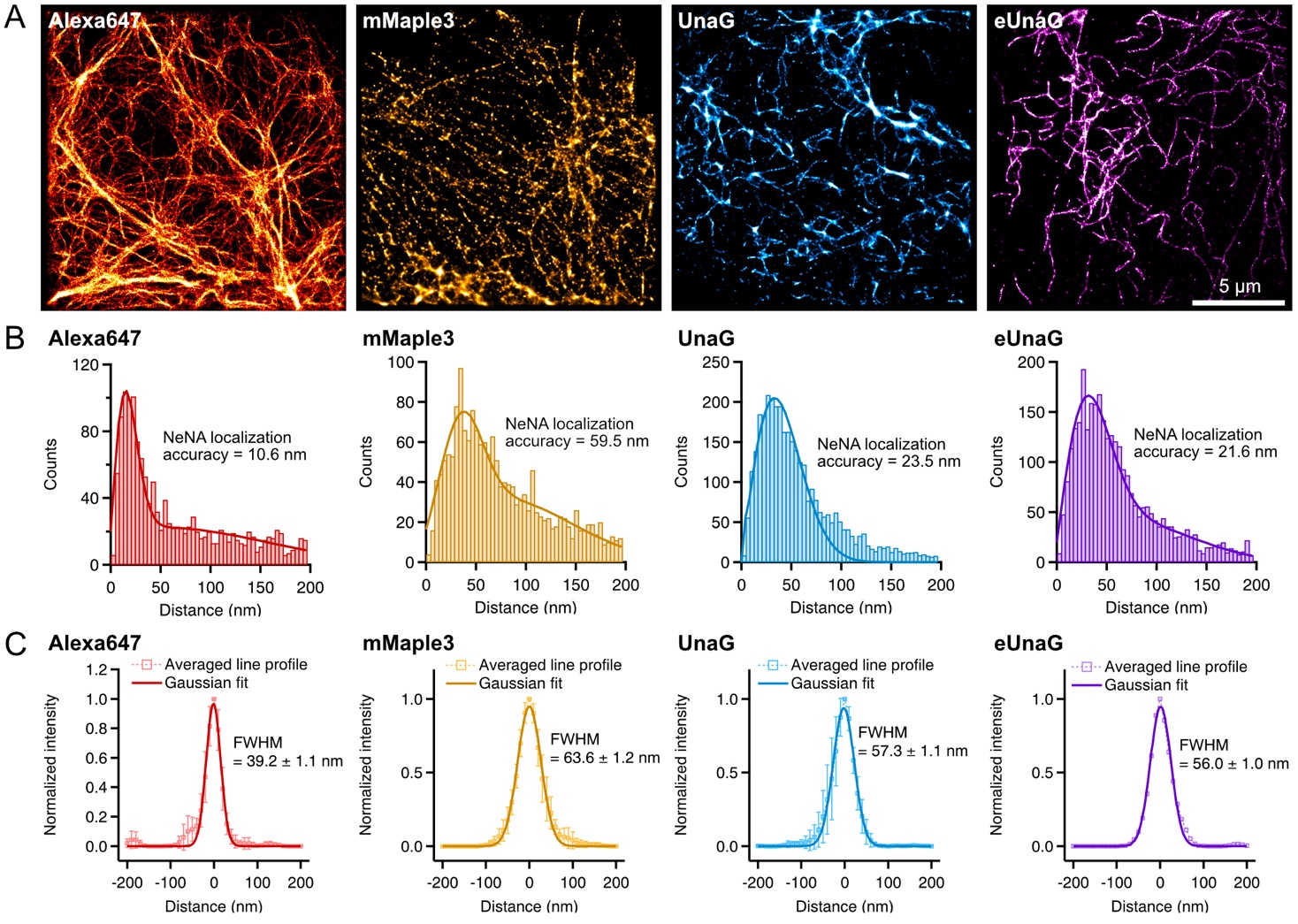


**Supplementary Figure 9.** Comparison of SMLM images of vimentin structures labeled with UnaG (blue) and eUnaG (purple) to those of Alexa 647 (red) and mMaple3 (orange). Alexa 647 image was obtained from vimentin fused with AviTag, a 12-amino-acid peptide tag that was biotinylated by biotin ligase enzymes and subsequently labeled with Alexa 647-conjugated streptavidin. All SMLM images were obtained without HAWK pre-processing. (**A**) Example SMLM images of vimentin structures. (**B**) Nearest neighbor-based analysis (NeNA) of the SMLM images in **A** giving localization accuracies indicated in the plots. Note that the NeNA localization accuracy of mMaple3 was noticeably larger than reported localization accuracies probably due to the low sampling coverage of the mMaple3-labeled fibrils. For high-density images of Alexa 647, UnaG and eUnaG, the NeNA localization accuracies were consistent to the reported values (i.e., ~10 nm for Alexa 647 and ~20 nm for EosFPs). Multiple molecules in close proximity detected as nearest neighbors of adjacent frames appear as non-Gaussian tails with large distances (Endesfelder et al., 2014). Thus, the smaller tails in UnaG and eUnaG NeNA distributions indicate that the on-off duty cycles were better controlled than the Alexa 647 and mMaple3 SMLM images. (**C**) Average line profiles of 5 vimentin filaments selected from SMLM images. The mean and standard deviation of FHWM values are indicated in the plots. Note that the eUnaG FWHM result in this plot obtained without HAWK pre-processing is similar to that in Supplementary Figure 9 with HAWK analysis.

**Supplementary Figure 10.** Analysis pipeline for the sampling coverage. Each vimentin filament was segmented manually and straightened in ImageJ. Each straightened image was binarized by using a custom MatLab code, and the sampling coverage was calculated from the middle three pixels of the binarized image.


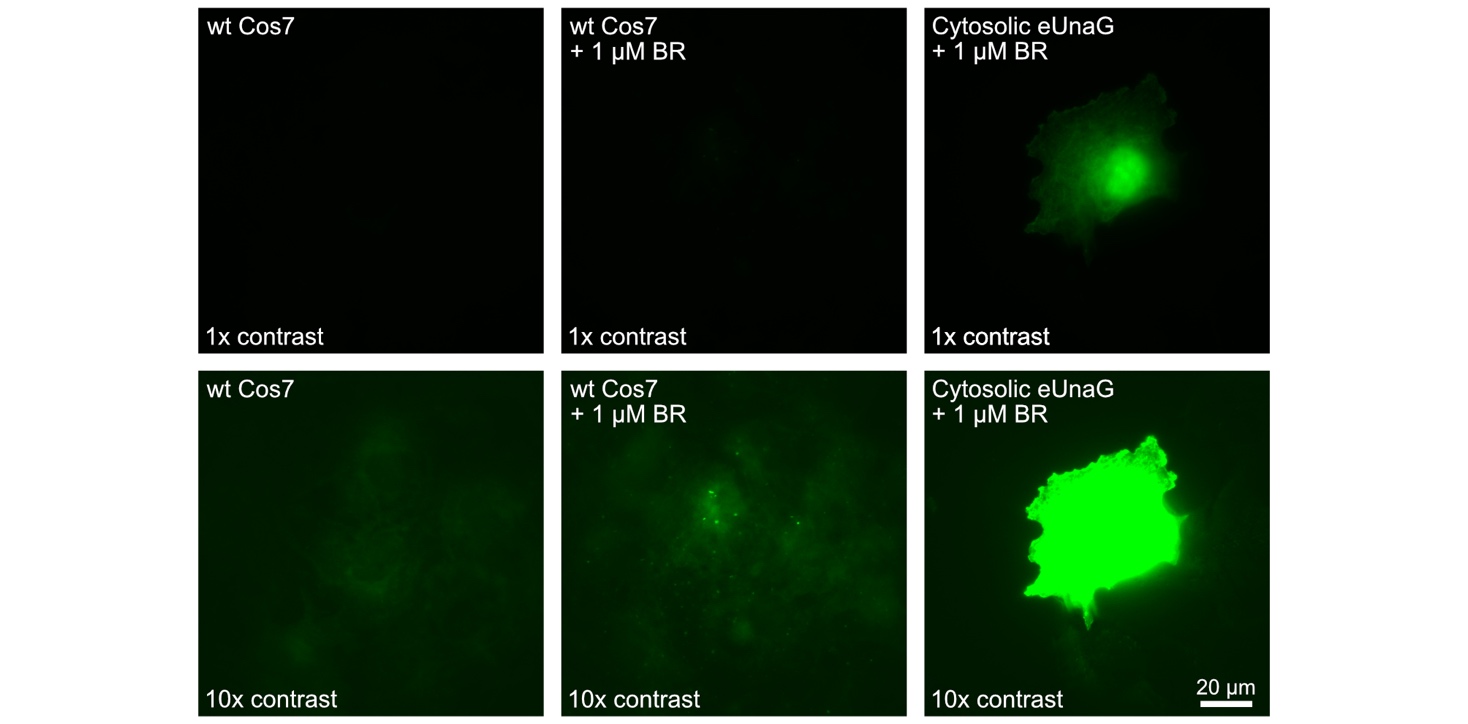


**Supplementary Figure 11.** Non-specific background fluorescence of bilirubin ligand. Epi-fluorescence images were obtained from wild-type cells without (left) and with bilirubin (center) in imaging buffer as well as from eUnaG-expressing cells with supplemented bilirubin (right). The contrast was adjusted to eUnaG (top) and autofluorescence (bottom). The background fluorescence of bilirubin was slightly higher than those of autofluorescence, but was negligible when compared to eUnaG fluorescence.


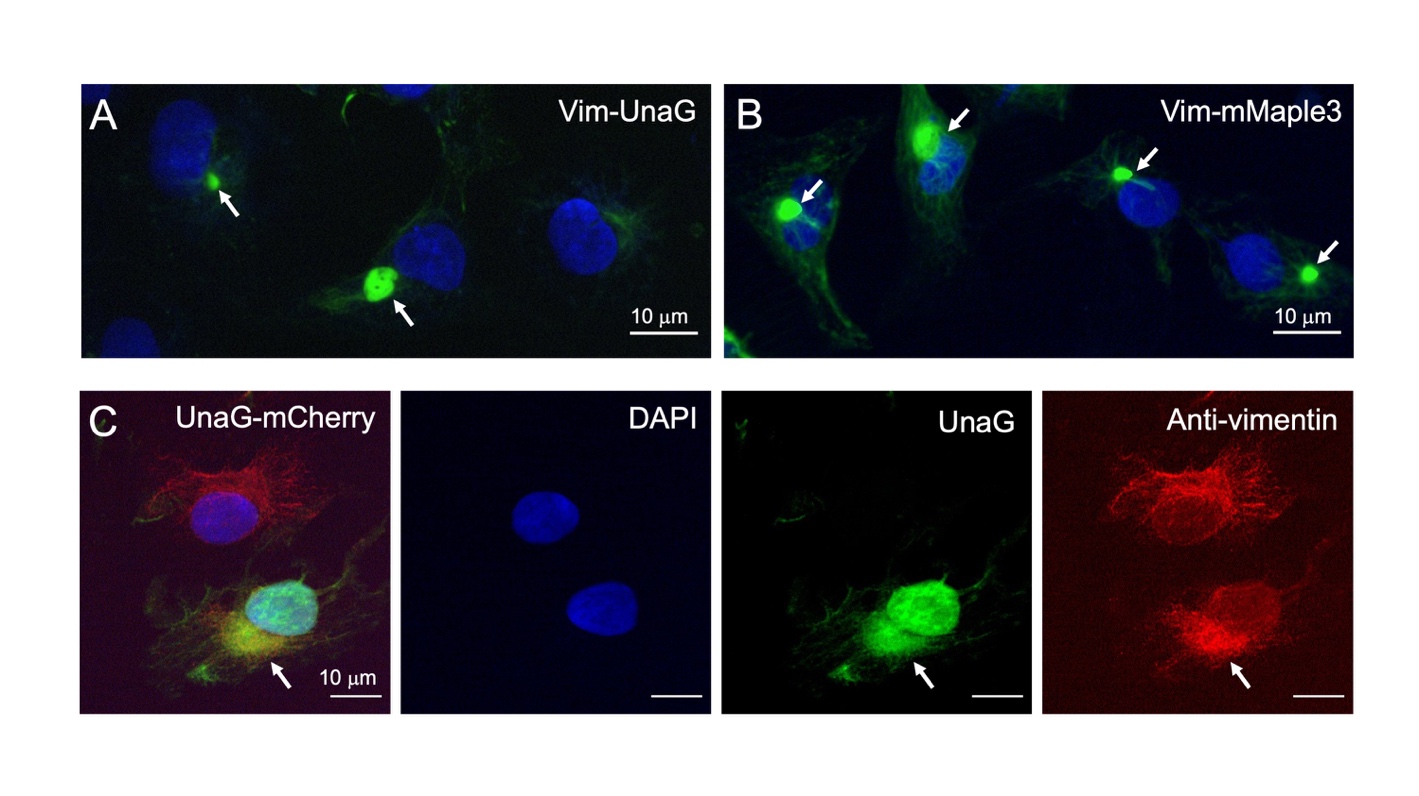


**Supplementary Figure 12.** Vimentin patches found in cells transfected with Vim-UnaG (**A**), Vim-mMaple3 (**B**) and UnaG-mCherry (**C**). (**A**,**B**) Example confocal images of cells transfected with Vim-UnaG or Vim-mMaple3 (green) and stained with DAPI (blue). In some cells expressing vimentin fused with UnaG (**A**) or mMaple3 (**B**), bright circular vimentin patches (white arrows) were found in the perinuclear region, consistent to typical vimentin structures associated with the aggresome. (**C**) Example confocal images of Cos-7 cells transfected with UnaG-mCherry and immunostained with a primary antibody for vimentin (Thermo Fisher, MA5-11883) and a secondary antibody conjugated with Alexa 647 (red). A small fraction of cells expressing UnaG (green) contained an aggregate of UnaG (white arrows), co-localized with a patch of endogenous vimentin, in the perinuclear region. Since the UnaG-mCherry plasmid does not contain any targeting sequence, most cells expressing UnaG-mCherry showed uniform cytosolic fluorescence. Also, cells without UnaG-mCherry fluorescence (e.g., the cell in the top of the image) showed distributed vimentin fibrils. In contrast, the cell with a UnaG patch in the bottom showed vimentin concentrated on the aggregate as in aggresome-associated vimentin cages (Johnston et al., 1998).
